# Barriers to colonoscopy in UK colorectal cancer screening programmes: Qualitative interviews with ethnic minority groups

**DOI:** 10.1002/pon.6123

**Published:** 2023-04-06

**Authors:** Robert S. Kerrison, Natalie Gil, Elizabeth Travis, Robyn Jones, Katriina L. Whitaker, Colin Rees, Stephen Duffy, Christian von Wagner

**Affiliations:** ^1^ School of Health Sciences University of Surrey Surrey UK; ^2^ School of Psychology University of Leeds Leeds UK; ^3^ Department of Behavioural Science and Health University College London London UK; ^4^ Faculty of Medical Sciences Newcastle University Newcastle UK; ^5^ Wolfson Institute of Population Health Queen Mary University London London UK

**Keywords:** barriers and facilitators, colonoscopy, colorectal cancer, ethnic inequalities, ethnic Minorities, interviews, psycho‐oncology, qualitative research, screening

## Abstract

**Objective:**

People from ethnic minority backgrounds are less likely to attend colonoscopy, following faecal immunochemical test screening, and are more likely to be diagnosed with colorectal cancer at an advanced stage as a result. The aim of this research was to explore the barriers and facilitators to attending colonoscopy, perceived by ethnic minority groups living in the United Kingdom.

**Methods:**

Semi‐structured online and telephone interviews were conducted with thirty men and women of Black‐African (*n* = 5), Black‐Caribbean (*n* = 5), South Asian (*n* = 10) and White British (*n* = 10) descent. Participants were eligible for screening, but had not necessarily been invited for colonoscopy. All interviews were conducted in the participant's first language and were assessed using Framework‐analysis, in line with a conceptual framework developed from previous interviews with healthcare professionals.

**Results:**

Five thematic groups of barriers and facilitators were developed: ‘*Locus of control’,* ‘*Cultural attitudes and beliefs’*, *‘Individual beliefs, knowledge and personal experiences with colonoscopy and cancer’*, ‘*Reliance on family and friends’* and ‘*Health concerns’*. Differences were observed, between ethnic groups, for: ‘*Locus of control’*, ‘*Cultural attitudes and beliefs’* and ‘*Reliance on family and friends’.* Black and South Asian participants frequently described the decision to attend colonoscopy as lying with ‘God’ (Muslims, specifically), ‘the doctor’, or ‘family’ (*Locus of control*). Black and South Asian participants also reported relying on friends and family for ‘language, transport and emotional support’ (*Reliance on family and friends*). Black‐African participants, specifically, described cancer as ‘socially taboo’ (*Cultural attitudes and beliefs*).

**Conclusions:**

The results highlight several targets for culturally‐tailored interventions to make colonoscopy more equitable.

## 1 BACKGROUND

Colorectal cancer (CRC) is a leading cause of morbidity and mortality in Europe.^1^ Several large randomised controlled trials have shown that regular faecal immunochemical test (FIT) screening, between the ages of 45 and 80, can significantly reduce CRC‐mortality among people who complete the test.^2^ As a result, most European countries have implemented FIT‐based screening programmes for the early detection of CRC.^3^

As with all screening, the extent to which the benefits of FIT are realised, and the extent to which they are equitable, is highly dependent on uptake of the test, as well as any necessary follow‐up investigations (colonoscopy being the gold standard for FIT screening).^4^ However, in a recent international survey of 35 FIT‐based screening programmes, Selby and colleagues (2021) found that the mean proportion of participants, with a positive FIT result, who attend colonoscopy, was 79%, with completion rates ranging from 39% in the programme with the lowest level of colonoscopy attendance, to 100% in the country with the highest.^5^

As with uptake of FIT, evidence suggests that attendance at colonoscopy is lower among those from an ethnic minority background, compared with those of White British ethnicity.^6^ Such disparities may contribute toward ethnic inequalities in CRC outcomes seen in the UK.^7^ For example, Black and South Asian adults, living in the UK, are more likely to have lymph node involvement at diagnosis (compared with White adults),^7^ and are less likely to be diagnosed via screening (the diagnostic route associated with the best outcomes for CRC).^8^

To date, the majority of qualitative research exploring non‐attendance at colonoscopy has been conducted with White, English‐speaking, adults.^9^ The little research that has been conducted with non‐White populations has been conducted primarily in the USA, with Black African Americans.^9^ Key findings from a recent review of the literature indicate that procedural costs, perceived threat to masculinity and lack of insurance are among the most prevalent barriers to colonoscopy. However, the findings from these studies are not necessarily transferrable to the UK and other European countries, because of cultural differences between countries, differences in healthcare delivery, and the fact that colonoscopy is often offered as the primary screening test in the USA.^9^

The aim of this research, therefore, was to explore the perceived barriers and facilitators of colonoscopy, among ethnic minority groups (including native speakers of English and patients with limited English proficiency) living in the United Kingdom (UK).

## 2 METHODS

**Setting.** The study was conducted in the UK, where FIT‐screening, and colonoscopy (where required), are free at the point of use (FIT‐screening is offered through the National Health Service [NHS], as part of an organised National Bowel Cancer Screening Programme, with invitations delivered biennially, between the ages of 60 and 74 in England and Northern Ireland, 55–74 in Wales, and 50–74 in Scotland).

According to the most recent census (a national survey of UK households, undertaken by the Office of National Statistics), 9.3% of the population in England and Wales identify as ‘Asian, Asian British or Asian Welsh’ and 2.5% identify as ‘Black, Black British, Black Welsh, African or Caribbean’ (the census defines ethnicity as “The ethnic group that the person completing the census feels they belong to […] based on their culture, family background, identity or physical appearance”),^10^ making these two of the most common ethnic minority groups in the UK (data for and Wales Scotland and Ireland have not yet been published, and so the ethnic composition for these regions is not currently known; however, England and Wales account for 92% of the UK population, and thus reflect the majority of the population).^10^

The overall attendance at colonoscopy, within the screening programme, and across the population, is 80%.

**Study design and participants.** Online and telephone interviews were conducted with members of the British public, who: (1) were of screening eligible age in England, Wales, Scotland and Northern Ireland (i.e. aged 60–74 years), (2) had been invited to participate in bowel cancer screening at least once, and 3) were of either a South Asian, Black African, Black Caribbean or White British ethnic background (these ethnic groups were selected for inclusion in the research, on the basis that they are the least likely to attend colonoscopy and receive a diagnosis through the bowel cancer screening programme [with the exception of the White British group, which was included to help disentangle ‘ethnic group‐specific barriers’ to colonoscopy from ‘universal barriers’]). Non‐attendance at colonoscopy was not a requirement for inclusion in the study, as previous research has found that those who decline colonoscopy are unwilling to participate in such interviews.^11^ Participants were subsequently those who were characteristically unlikely to attend colonoscopy, as opposed to those who have been invited for (and not attended) colonoscopy. Key characteristics of participants (e.g. age, gender, ethnicity first language, etc.) were determined through a short survey, administered at the beginning of the interview (Appendix A).

**Sample size and recruitment strategy.** To minimise language barriers to participation, targeted recruitment strategies were employed, with White British participants being recruited via Social Media, and Black and South Asian participants being recruited through Agroni: a multi‐disciplinary research organisation with extensive specialist knowledge and experience working with Britain's ethnic minority group communities.^12^

Participants recruited through Agroni were identified and recruited by a team of professional recruiters, who visited community centres, Mosques, Mandirs, and charities, with which they had existing connections. Individuals who appeared to meet the eligibility criteria were approached by the recruiters, who discussed the study with them on a one‐to‐one basis (due to the sensitive nature of the topic; i.e. cancer). Those who expressed an interest in taking part in the study were given Agroni's contact details, and asked to call or email for further information about the research. All but one person given Agroni's contact details went on to complete an interview (i.e. 20/21).

Twitter and Facebook were used to recruit participants via social media. A digital flyer was used to provide details about the research and invite participants to call or email the lead researcher (RK), if interested in taking part.

A stratified sampling approach was used to ensure equal numbers of male and female participants, as well as participants of different ethnicities (the researchers had no prior relationship with the participants). The recruitment strategy also aimed to sample participants from across the UK, but was not stratified by region or nationality.

On the basis that data saturation is achieved after 9–17 interviews,^13^ we aimed to recruit 10 participants from each ethnic group (i.e. Black, South Asian and White British). Data saturation was subsequently assessed by reviewing whether new codes were developed following analysis of the 10th interview of each group.

**Data collection.** Interviews took place between July 2021 and July 2022. To minimise barriers to disclosing culturally sensitive information, interviews were concordant for gender, ethnicity and the participant's first language. RK (White male) conducted all interviews with White British participants, who identified as male (*n* = 5). NG (White female), meanwhile, conducted all interviews with White British participants, who identified as female (*n* = 5). Finally, Agroni researchers conducted all interviews with Black and South Asian participants, identifying as either male (*n* = 10) or female (*n* = 10). Zero participants identified as any other gender (e.g. non‐binary).

To ensure Agroni researchers understood the aims of the study, and thus conducted the interviews appropriately, RK met with them prior to data collection, to discuss the interview schedule and address any questions about the research. No additional training was given to Agroni researchers, as they were all experienced researchers with prior experience conducting qualitative interviews.

Interviews lasted 36 min on average (range: 24—67 min) and were conducted using a semi‐structured interview guide, which was developed by three members of the research team (RK, CvW and KW), all of whom have PhDs in Psychology. The interview schedule was developed using a conceptual framework, which was developed through interviews with specialist screening practitioners in a previous study led by the research team.^14^ The interview schedule was pilot tested with two individuals (one by RK, with an individual of White British ethnicity; one by Agroni, with an individual of South Asian ethnicity), prior to data collection, to ensure participant comprehension of the questions (no changes were made to the interview guide). Questions focussed on patients' perceived barriers and facilitators to colonoscopy attendance, following participation in a FIT‐based screening programme for CRC (see Appendix B).

An audio recorder was used to record the interviews. The recordings were anonymized, translated and transcribed verbatim by K International: a UK‐based translation and transcription services provider.^15^ Following transcription, the audio files were deleted by K International and the research team.

Participants were given the option to participate in either an online or telephone interview. Participants who opted to participate in an online interview were given a choice of MS Teams or Zoom. All participants opting for an online interview opted to participate via MS Teams. The interviews were conducted remotely, from the researchers' and participants' homes. No one was present during the interviews, besides the researcher and the participant.

**Informed consent.** Informed consent was obtained from participants, before the interviews commenced. For White British participants, an information sheet and consent form were provided, via email, to those expressing an interest in taking part. A mutually convenient date for interview was then agreed, for those who returned a completed consent form (via post or email). The purpose of the study, the right to withdraw from the study, and the right to skip any questions, were repeated at the beginning of the interview, along with the participant's decision to proceed with the interview.

For South Asian, Black African and Black Caribbean participants, Agroni researchers explained the purpose of the study to potential participants, in their first language, over the phone. For those interested in taking part, Agroni Researchers subsequently read through the information sheet and consent form (again, over the phone). Those who were interested in participating, after providing verbal consent, were offered an interview on a mutually agreeable date. As with the White British interviews, the purpose of the study, the right to withdraw from the study, and the right to skip any questions, were repeated at the beginning of the interview, along with the participant's decision to proceed with the interview.

**Data analysis.** Transcripts were analysed using framework analysis.^16^ This method is well‐suited for generating knowledge relevant for health and clinical practice,^17^ allowing for in‐depth understanding *within* individual cases, as well as analyses of key themes *across* the data‐set. While this is a flexible method, not aligned with a particular epistemological, philosophical, or theoretical approach, this study adopted an interpretivist approach, which recognises the importance of situating the researcher in the context of that which is being studied,^18^ to offer an interpretive understanding of the meaning participants ascribe to their own experiences.^19^ A detailed overview of the analysis process is provided below:

### 2.1 Initial development of codes (stages 1–4)

*Stage 1: Transcription*. Translation and transcription of the interviews was carried out (verbatim) by K International.

*Stage 2: Familiarisation with the interview data.* Transcripts were read and re‐read, and typed reflections of initial thoughts and observations were captured in the page margins by two researchers (RK and NG).

*Stage 3: Coding.* Codes were developed to help describe and classify the data in relation to the research question (i.e. segments of text were assigned codes that reflected the issues discussed within those segments; e.g. ‘Importance of female healthcare professionals’). Two researchers (RK and NG) initially independently developed ideas for codes using the same sample of transcripts (*n* = 6; 20%). Codes were generated inductively (i.e., from the data) and deductively, according to a framework previously developed from interviews with specialist screening practitioners, led by the research team (i.e., codes were used/adapted from a list of previously curated codes, as and when relevant to the data; see Appendix C).^14^

*Stage 4: Development of a working analytical framework.* Researchers (RK and NG) met to compare their independently generated ideas for new and revised codes and, through discussion, consensually agreed on a working analytical framework that could be applied to further transcripts (i.e., a ‘revised’ set of codes and the meaning of each).

### 2.2 Application of codes (Stage 5)

*Stage 5: Applying the analytical framework.* One researcher (NG) then coded the remaining transcripts using the revised codes. Where a new transcript was judged to contain text that could not be satisfactorily coded using the existing codes, new codes were created, or existing codes updated to more accurately reflect the data they represented. Any new codes were discussed and agreed with RK, before NG subsequently revisited previously coded transcripts to apply the new codes (if/where relevant).

### 2.3 Use of coded material to develop themes (Stages 6 and 7)

*Stage 6: Charting data into the framework matrix.* The data were charted into a matrix, to provide a summary of the transcript material assigned to each code per participant (some cells were blank where no material existed in a transcript for a particular code; see: https://osf.io/pvk3w/). The charted framework matrix was then reviewed by three researchers (RK, NG and ET) to develop themes (all stages of coding and data analysis were carried out in Microsoft Excel).

*Stage 7: Interpreting the data.* Themes were developed to delineate key messages in the data, relevant to the research aims. Data interpretation involved making comparisons between the barriers and facilitators reported by participants, according to their ethnicity and religion. Theme development was iterative and involved all researchers, who reached a consensus through discussion on the final content and organisation of themes.

**Data saturation.** Data saturation was assessed for each ethnic group, using a data saturation matrix (see: https://osf.io/pvk3w/). No new codes were developed after the 8th interview with White British participants, or the 9th interview with South Asian participants, and only one new code was developed during the tenth and final interview with Black participants.

**Transparency.** This study has been reported in accordance with the Consolidated Criteria for Reporting Qualitative Research (COREQ) guidelines (Appendix D).^20^ A database of the coded text, along with an audit trail, is available from Open Science Framework, for full transparency (see: https://osf.io/pvk3w/).

**Ethics.** The study was approved by University College London's Joint Research Office (reference: 5299/003) on the 19th of May 2021. NHS Research Ethics Committee approval was not required for the research, as participants were members of the public, not patients.

## 3 RESULTS

### 3.1 Participant characteristics and data collection

In total, 15 men and 15 women participated in the study (none of whom dropped‐out or withdrew from the research). Participants were of a range of ethnicities, including White British (*n* = 10), South Asian (Pakistani, *n* = 2; Indian, *n* = 4; Bangladeshi *n* = 4) and Black African and Caribbean (Black African, *n* = 5; Black Caribbean, *n* = 5). The majority of participants were from London (*n* = 18), with just under half being from other regions (*n* = 12). Participants were interviewed in a range of languages, including English (*n* = 15), Somali (*n* = 5), Bengali (*n* = 4), Punjabi (*n* = 3), Urdu (*n* = 3) and Guajarati (*n* = 1). Participants were also from a range of religions, including Islam (*n* = 11), Christianity (*n* = 8), Sikhism (*n* = 3) and Hinduism (*n* = 1). All had been invited for bowel cancer screening (*n* = 30), although less than half had ever taken part (*n* = 13). Twelve discussed having had at least one colonoscopy during their lifetime. Aggregate participant characteristics are summarised in Table 1; individual‐level characteristics are reported in the appendix (see Appendix E).

**TABLE 1:** Sample characteristics.

Age (years)	
Mean	66.1
Gender (*n*)	
Male	15
Female	15
Region (*n*)	
London	18
Birmingham	2
Leicester	2
Oldham	1
Nottingham	1
Gateshead	1
Surbiton	1
Wiltshire	1
Clacton‐on‐Sea	1
Horsham	1
Glasgow	1
Ethnic group (*n*)	
White British	10
Black African (Black)	5
Black Caribbean (Black)	5
Bangladeshi (South Asian)	4
Indian (South Asian)	4
Pakistani (South Asian)	2
First language/language the interview was conducted in (*n*)	
English	15
Somali	5
Bengali	4
Punjabi	3
Urdu	2
Guajarati	1
Religion (*n*)	
Islam	11
Christianity	8
Sikhism	3
None	3
Missing	3
Jehovah's Witnesses	1
Hinduism	1
Previously invited for bowel cancer screening (*n*)	
Yes	30
No	0
Previously participated in bowel cancer screening (*n*)	
Yes	13
No	17
Previously received colonoscopy (*n*)	
Yes	12
No	18

### 3.2 Description of themes

Five key themes were developed, namely: (1) *Locus of control, (2) Cultural attitudes and beliefs, (3) Individual beliefs, knowledge and personal experiences with colonoscopy and cancer*, *(4) Reliance on family and friends* and *(5) Health concerns.* A detailed overview for each, and their constituent subthemes, follows. Example quotes for all subthemes are presented in Table 2. A summary of the themes, subthemes and codes developed is presented in Table 3.

1. **Theme one.** Locus of control: the role (or influence) of others in the decision making process was a central theme of the research, with individuals frequently discussing the extent to which it is others, not themselves, who are in control over the decision to attend colonoscopy.
1.1 (The role of) The medical professional, the patient and the patient's family in the decision making process

**TABLE 2:** Example quotes.

Subtheme	White British	South Asian	Black
Theme 1. Locus of control: The role (or influence) of others in the patient's decision making process			
1.1. (The role of) The medical professional, the patient and the patient's family in the decision making process	Participant 23 (White British, female). “*I certainly looked up online what colonoscopy entailed and what would happen and things like that. Maybe not about bowel cancer, as such, but about the procedure.*”	Participant 1 (Bangladeshi, male). “*Whatever you have, you must go for a check‐up. We do not have any other option. We must follow the doctor's instructions*"	Participant 21 (Somali, male). “*Yes (I would attend) because the appointment is from a doctor and I can't refuse, so I have to accept*”
		Participant 2 (Bangladeshi, female). “*If I face such a situation, I would first show it to my children. If it relates to medicine, I'll show it to <Name>, who is a medical student working at a hospital. She is my eldest daughter. Whenever I felt an issue, I always asked her for assistance. Recently, I had an infection in my urine. I asked her for assistance. She told me, "Mom, don't do this, do that*"	Participant 12 (Black Caribbean, female). “*Yeah, my daughter would say, ‘mum, if it's going to help’, right. Well, one of them would say, ‘mum, if it's going to help, right, just do it’, you know what I mean? And then it would be up to me, isn't it?*”
1.2. (The influence of) God and religion		Participant 10 (Pakistani, female). “*No one can change the decision of God, not the doctor, nor any other physician.*”	Participant 20 (Somali, female). “*I would have just say I put all my trust in God and go to the appointment*”
Theme 2. Cultural attitudes and beliefs			
2.1. Cultural taboos	Participant 29 (White British, male). “*I mean, you know, we…it's one of those, erm, social taboos, isn't it? Generally, we don't talk about poo, erm, and, err, we need to, really. It's fairly obvious to me that, in terms of, erm, you know…people not knowing what's normal, like, in terms of how often you poo, and what your poo looks like, it is a bit of a, erm, social embarrassing area*”	Participant 5 (Indian, female). “*My friends are old like me, so I cannot discuss with them. My daughter‐in‐law organizes everything for me, so I will discuss it with her*”	Participant 17 (Somali, male). “*Our culture is very sensitive to the problem. This is another challenge. The Somali people are so scared about it (cancer) and that is another big challenge and the issue is so sensitive culturally and in addition the disease has created fear to the people*”
			Participant 21 (Somali, male). “*But the Somali people believes that it is ashamed and they don't want to be removed from the clothes. Would they do that to me? They believe that is a shame. But it is not, when you're working on health*”
	Participant 25 (White British, female). “*I hope you manage to get more people turning up for it, because you know yeah it's, it's bums isn't it*”		
			Participant 21 (Somali, male). “*She said that ‘I can't accept such thing to be said to me’. I was so shocked to hear that. She said that ‘what are you saying to me such evil thing. How can you say we will put something in your ass and it will checked whether you have cancer or not’. They don't want someone to see their private organs*”
2.2. Importance of Female HCPs			Participant 21 (Somali, female). “*We are Muslims a man should not test us […] I told them, I will not be tested by a man*”
Theme 3. Individual beliefs, knowledge and personal experiences with colonoscopy and cancer (Personal experiences and knowledge)			
3.1. Individual beliefs about colonoscopy and cancer as a treatable disease (Fatalism)	Participant 22 (White British, male). “*Better to have any test, go through any discomfort, but that if you have got cancer, you can catch it as early as possible, because the earlier you can detect it the more chance there is of getting rid of it*”	Participant 01, South Asian, gender. “*There is no need to worry because the technology is so advanced now. If it is not positive then it is best but if we get a positive result, then we have to consult with the doctor*”	Participant 12 (Black Caribbean, female). “A*s I said to my kids, if I'm going to die, I'm going to die. If it's my time, it's my time and I don't want no one cutting me up and slice and dicing me*”
3.2. Fear of cancer	Participant 26 (White British, female). “*I had some bleeding a couple of years ago. I was there in a flash because it frightened the life out of me*”	Participant 1 (Bangladeshi, male). “*Usually people think that they have cancer and that's why they want to be examined like this.*”	Participant 14 (Black Caribbean, male). “*To find out if my body is fit and if it's not fit I try to go and get it… bowel cancer and all those things*”
	Participant 31 (White British, male). “*I've known a lot of people who have chosen not to go ahead because they didn't want the answer. You know they're almost frightened of the answer*”		
3.3. Knowledge of bowel cancer and the risks and benefits of colonoscopy	Participant 29 (White British, male). “*Just that the problem is not going to go away, so you need to do something about it and that early treatment is best in terms of early treatment options available to you and the best options*”	Participant 8 (Indian, female). “*I'm just worried about this bleeding thing only, yeah, for that. It's not the… even a small cut can make it, you know…*”	Participant 15 (Black Caribbean, male). “*It is important because if you find out that you have any growths they can remove it in time, before it turns to cancer. So it would be good for you to know in time*”
3.4. Personal experiences and experiences of others with colonoscopy and cancer	Participant 27 (White British, male). “*Yeah, I mean, the sort of the report, if you like, after the operation, they said, you know, ‘we did this, we did that’, um and ‘we are confident’ and, not that they used that word, but they were confident that they had, um, you know, removed all the cancerous tissue, I suppose. And they, and I was now on this five year watch programme, um, but you know, when I've been in November, and they say ‘yeah this all looks good’, when I've had a look myself, which is, as I say, the interesting part, and, uh, they say ‘yeah there's nothing, this all looks good to us' then, you know, that puts your mind at rest, sort of thing. So yeah, I'm not looking forward to it, but I'm looking forward to it. If you know what I mean?*”	Participant 10 (Pakistani, female). “*I have experience with colonoscopy, and I am not afraid of it.*”	Participant 21 (Somali, male). “*I have gone and they have entered my body with the colonoscopy. They told me you're okay and your negative. But that just reassured me, and it reassured me*”
	Participant 29 (White British, male). “*Erm, yeah, it wasn't a great experience. Erm, it was a bit more painful than I thought it was going to be […] I could definitely say, yeah, if it had to be done again tomorrow, yes, I'd say, ‘oh God, here we go again, let's do it’”*		
	Participant 29 (White British, male). “*As I say, I had my cancer screening before my dad died, but now, on account of my dad's death, yes I would be exceptionally keen…exceptionally keen to get it*”		
3.5. Lack of trust in western medicine			Participant 12 (Black Caribbean, female). “*When I first did the first screening, because I do eat a lot of pepper, they came back and they said that I had blood in my stool, and I thought, you know what, you're just trying to flipping get me in your bosoms. Me, I don't trust them, and I never will*”
3.6. Valuing health	Participant 22 (White British, female). “*Yeah you just can't put anything off. I mean if I get any lumps even if it's my glands under my arms or any lumps, I'm straight down the doctor*”	Participant 10 (Pakistani, female). “*Whether it is cheap or expensive, we have to do everything for the betterment of our health”*	Participant 18 (Somali, male). “*My health is always my first priority*”
Theme 4. Reliance on family and friends.			
4.1. Reliance on family and friends as unofficial interpreters		Participant 5 (Indian, female). “*It is not that they do not go because they are from India or Pakistan. But sometimes they do not have anyone who can read this letter for them*”	Participant 20 (Somali, female). “*The number of the interpreters are few […] Therefore, for me to be at least aware of my healthy status perfectly, I go with someone*”
			Participant 20 (Somali, female). “*Yes, it is the language. They don't know the language and that is killing them and making them suffer*”
4.2. Reliance on family and friends for transport	Participant 22 (White British, male). “*I would take my husband [to colonoscopy appointment] so he could drive back or he could drive because I know it's uncomfortable afterwards*”	Participant 7 (Indian, male)“*I will go with my children, or maybe with my wife. I am not allowed to go anywhere alone*”	Participant 13 (Black Caribbean, Female). “*I have a problem with my back, I can't stand straight, so more time, I have to ask my daughter and she will take me there*”
4.3. Reliance on family and friends for emotional support		Participant 9 (Pakistani, female). “*I will go with someone else. It will be easier for me. It encourages us that I have somebody with me*”	Participant 19 (Somali, male). “*I would go with someone else. Because of fear. One of my family members. Either my nephew or my lady or my wife. I would have gone with a family member because it is a disease and you need help since it worries you a lot*”
Theme 5. Health concerns			
5.1. Existing health conditions	Participant 29 (White British, male). “*If it was clashing with erm, having another medical appointment that is going to have a higher priority […] I've had a brain haemorrhage in the past. If they were to ring up and say ‘<Name> I think your brain haemorrhage is back again’, I might give that priority*”	Participant 8 (Indian, female). “*It is just because of this… my blood too thin and sometimes if I get a cut or anything, it can bleed, yeah. So the doctors… so this is what the worrying thing, is that, I should not have any cut or bruise or anything or any scratch. It will start bleeding and will not stop, yeah*”	Participant 13 (Black Caribbean, Female). “*I have osteoarthritis and I hurt my spine. I have diabetes. I have high blood pressure. I'm not good and I do one knee replacement already [inaudible 00:14:48] and I have to do the other one, but I'm waiting on the appointment to go to the hospital. I would like to know how I am going to get through it, what the procedure, what they're going to do; things like that*”
5.2. COVID	Participant 24 (White British, female). “*I mean I haven't had Covid‐19, amazingly. Um, but no, I would still feel happy to go, assuming that everybody was wearing a mask, and taking precautions, and had been vaccinated*”	Participant 7 (Indian, female). “*Covid‐19 is not an issue at all. I have already received two doses of Covid‐19 vaccine*”	Participant 18 (Somali, male). “*It is very good to be careful. Disease [COVID‐19] is rampant in the world*”
		Participant 3 (Bangladeshi, male). “*Because of the pandemic, I cannot go anywhere*”	
		Participant 5 (Indian, female). “*I used to attend before Covid‐19, but I did not attend any community group after Covid‐19. I do not go outside*”	

**TABLE 3:** Summary of the themes, subthemes and codes identified.

Theme	Subtheme	Code
1. Locus of control: The role (or influence) of others in the patient's decision making process	1.1. (The role of) The medical professional, the patient and the patient's family in the decision making process	1.1.1. Free will/personal choice in medical decision making
		1.1.2. Obtaining detailed information facilitates participation
		1.1.3. Reliance on medical professional/authority
		1.1.4. Shared decision making and family influenced participation
	1.2. (The influence of) God and religion	1.2.1. Religious faith facilitates participation
		1.2.2. The role of God in determining the future
2. Cultural attitudes and beliefs	2.1. Cultural taboos	2.1.1. Cultural taboos
	2.2. Importance of female HCPs	2.2.1. Importance of female HCPs
3. Individual beliefs, knowledge and personal experiences with colonoscopy and cancer	3.1. Individual beliefs about coloscopy and cancer	3.1.1. Perception of colonoscopy as life‐saving
		3.1.2. Belief that cancer is a treatable disease
	3.2. Fear of cancer	3.2.1. Fear of cancer
	3.3. Knowledge of bowel cancer and the risks and benefits of colonoscopy	3.3.1. (Lack of) Knowledge about CRC, screening and colonoscopy
		3.3.2. Concerns about perforations or procedural risks
		3.3.3. Perceived importance of screening
	3.4. Personal experiences and experiences of others with colonoscopy and cancer	3.4.1. Concerns about bowel prep
		3.4.2. Concerns about pain and discomfort
		3.4.3. Personal and family experiences with colonoscopy
		3.4.4. Peace of mind
		3.4.5. Personal or family history of bowel cancer
		3.4.6. Personal or family experience as a healthcare professional
		3.3.7. Attitudes towards free healthcare, regular health checks, healthcare professionals and healthcare provision in the UK
	3.5. Lack of trust in Western Medicine	3.5.1. Lack of trust in Western Medicine
	3.6. Valuing health	3.6.1. Valuing health
4. Reliance on family and friends	4.1. Reliance on family and friends as unofficial interpreters	4.1.1. Reliance on family and friends as unofficial interpreters
	4.2. Reliance on family and friends for travel & transport	4.2.1. Reliance on family for travel & transport
	4.3. Reliance on family and friends for emotional support	4.3.1. Reliance on family for emotional support
5. Health concerns	5.1. Existing conditions	5.1.1. Existing health conditions interfering with ability to complete procedure/acting as a competing priority
	5.2. COVID	5.2.1. Fear of COVID‐19
		5.2.2. Vaccination status and hygiene

There appeared to be differences, between ethnic groups, regarding where control over the decision‐making process lie. Black and South Asian participants frequently indicated that it was the medical professional's role to advise them what they should do to protect their health, and that it was not for the individual to challenge the doctor's advice.

In addition to discussing it as being primarily the doctor's decision, South Asian participants frequently reported that the family held an important role in the decision‐making process, and that they would follow the advice of their partners and children. Black participants also highlighted that they would discuss it with their family, but argued it was not the family's decision to make.

White British participants, meanwhile, predominantly discussed making the decision independently of others, and going online for information and advice to help them make their decision. This process was, for example, discussed by one patient, who had recently attended colonoscopy.

1.2. (The influence of) God and religion

South Asian and Black African adults, who were of Muslim faith, also frequently discussed God, saying they would “trust in God” and attend the appointment. Paradoxically, other South Asian and Black African participants, also of Muslim Faith, indicated they would not go, as “No one can change the decision of God, not the doctor, nor any other physician”.

White British adults, and those who were not of Muslim faith, meanwhile, did not discuss God, despite many of them being of Christian faith.

2. **Theme two.** Cultural attitudes and beliefs

Cultural attitudes and beliefs (religious and non‐religious) were also frequently discussed by participants, and included a broad range of cultural taboos, and the importance of having a female healthcare professional perform the examination.

2.1. Cultural taboos

Colonoscopy, colons, rectums and cancer were all discussed as culturally taboo topics by participants. For Black African participants, cancer, in particular, was reported to be culturally taboo and feared by the community. Black African participants also discussed the need to undress and have “something in your ass” (referring to the endoscope) as “evil” and “shameful” (these views were not discussed by Black Caribbean participants). South Asian participants, meanwhile, indicated that, while they could not discuss such issues as colonoscopy readily with their peers, they could (and would) discuss them with their family. For White British participants, however, it was “bums” and “bowel habits”, specifically, which were discussed as being socially taboo.

2.2. Importance of Female HCPs

Only one participant (Black African) highlighted the importance of having a same‐sex practitioner, specifically for Muslim women. Their views on this were very strong, however, with them stating: “we are Muslims, a man should not test us”.

3. **Theme three.** Individual beliefs, knowledge and personal experiences with colonoscopy and cancer

In addition to cultural attitudes and beliefs, which were shared by individuals of the same ethnic or religious group, individual beliefs, knowledge and personal experiences with colonoscopy and cancer were identified as barriers and facilitators to attending colonoscopy, and appeared to be unrelated to ethnicity and religion.

3.1. Individual beliefs about colonoscopy and cancer as a treatable disease

One of the most frequently discussed beliefs was that cancer is, or is not, a treatable disease (fatalism). Those holding the view that cancer is treatable, often described this as a reason for attending colonoscopy, while those holding the view that it is not treatable, described it as a reason for not attending.

3.2. Fear of cancer

While cancer was not discussed as a culturally taboo topic for non‐Black African participants, fear of cancer was discussed as both a barrier and a facilitator to attending colonoscopy, by participants of all ethnic groups (fear of cancer was different from fatalistic beliefs, in that it did not necessarily relate to dying, but the physical and emotional impact of receiving a cancer diagnosis, receiving treatment, etc.).

3.3. Knowledge of bowel cancer and the risks and benefits of colonoscopy

People's level of knowledge about bowel cancer and colonoscopy also presented as both a barrier and facilitator to attending colonoscopy. Knowing the risks associated with the colonoscopy procedure, for example, was reported as a barrier, while knowing that the test can help prevent cancer, through the removal of pre‐cancerous growths, was reported as a facilitator by others.

3.4. Personal experiences and experiences of others with colonoscopy and cancer

Participants, of all ethnic groups, frequently discussed their personal experiences with colonoscopy. These experiences generally manifested as facilitators for attending potential future colonoscopies, even when the test was described as “painful”, as they demystified the procedure. Those who previously attended colonoscopy also discussed the peace of mind it provided, and referred to this as an important motivation for attending future colonoscopies. The same motivator was reported as a reason for going to colonoscopy, by those who had not yet been invited. Those with a family history of bowel cancer, in particular, endorsed attending/wanting to attend colonoscopy, for this reassurance.

3.5. Lack of trust in western medicine

One Black Caribbean participant, who had previously had a positive bowel cancer screening test result, reported that they did not attend colonoscopy, as they “did not trust Western medicine”. As only one participant endorsed this view, and was not discussed in relation it to their ethnic background, or religious beliefs, in any way, it was not possible to attribute this belief to their cultural background.

3.6. Valuing health

Participants from all ethnic groups highlighted the value they place on their health, and discussed the importance of doing “everything for the betterment of our health” as a reason for attending colonoscopy and other healthcare appointments.

4. **Theme four.** Reliance on family and friends.

Many participants reported that they relied on friends and family when attending hospital appointments, and that the same was/would be true for attending colonoscopy. Participants relied on family and friends in a number of ways, including transport (getting to and from the hospital), translation services (interpreting the information materials, the nurses and doctors, etc.) and emotional support. While reliance on family and friends did not appear to be intrinsically linked to ethnicity, it was reported more frequently, and more prominently, by Black and South Asian participants.

4.1. Reliance on family and friends as unofficial interpreters

Being unable to read English was described as a barrier to attending colonoscopy (by Black African and South Asian participants), one which could not be overcome without the help of friends and family. Being unable to speak English was also described as a barrier to attending colonoscopy (again, by Black and South Asian participants). Here, too, some participants described relying on the support of family members as interpreters, with some reporting that official interpreters were not always available (where they were available, however, participants stated they would use them to overcome language barriers).

4.2 Reliance on family and friends for transport

Several Black and South Asian participants also reported relying on family and friends to take them to the hospital. In one instance, this was because they were “not allowed to go anywhere alone”, while, in another, it was because they had health issues, making it difficult for them to attend by themselves. One White British participant, who had previously attended colonoscopy, indicated that they would need support from friends and family to get to and from the hospital.

4.3 Reliance on family and friends for emotional support

Black and South Asian participants also reported wanting to bring a friend or family to their appointment for emotional support (while not overtly linked to ethnic group, the need for emotional support from others was not as prominent among the White British participants interviewed).

5. **Theme 5.** Health concerns

Finally, participants of all ethnic groups discussed pre‐existing health conditions, and the risk of getting COVID, as barriers to attending colonoscopy.

5.1 Existing health conditions

Several participants discussed existing health conditions as barriers to colonoscopy, either because they were higher priority, or because they presented possible complications (for example, one participant, who was taking warfarin, was worried about potential bleeding, while another, who had multiple morbidities, was concerned how the doctors would accommodate their physical condition).

5.2 COVID

Several participants also expressed concerns about COVID. Such concerns were expressed by participants from all ethnic groups, but were more prevalent and pertinent among those of Black and South Asian ethnic groups. Interestingly, others felt the pandemic was “over”, and that COVID was “not an issue”.

## 4 DISCUSSION

### 4.1 Summary of main findings

This study described five thematic groups of barriers and facilitators to attending colonoscopy among White, Black and South Asian adults, namely: (1) *Locus of control,* (2) *Individual beliefs, knowledge and personal experiences with colonoscopy and cancer*, (3) *Cultural attitudes and beliefs*, (4) *Reliance on family and friends* and (5) *Health concerns*. For several of these, there appeared to be differences between ethnic groups; this was particularly true for ‘*Locus of control’*, with Black and South Asian adults frequently describing the decision to attend colonoscopy as a decision made by the doctor, one prohibited/enabled by God (for Muslim participants), or one made jointly with the family. These views contrasted with those of White British participants, who frequently described the decision to attend colonoscopy as one they would make independently, through online research, or following discussion with friends, family and/or healthcare professionals.

There also appeared to be differences, between ethnic groups, with regards to ‘*Reliance on family and friends*’. Black and South Asian participants frequently discussed relying on family and friends throughout the colonoscopy process, including reading and understanding the invitation letter (with the exception of Black‐Caribbean participants), getting to and from the appointment, and interpreting what the nurse/doctor is saying during the consultation/appointment. White British participants, meanwhile discussed such issues to a much lesser extent.

Finally, there appeared to be differences, between ethnic groups, with regards to ‘Cultural attitudes and beliefs’. Black African participants, specifically, discussed cultural sensitivities around cancer, the need to undress for colonoscopy, and having to ‘put something in your ass’, describing each of these as ‘frightening’, ‘shameful’ and ‘evil’, respectively (these issues were not discussed by Black Caribbean participants). South Asian participants often discussed these matters as ‘private’, and indicated they would not discuss them with the wider community (although they would discuss them with their partners/children), while White British participants were more open to discussing such issues with a wider network of friends and family (particularly cancer), although they too regarded topics such as ‘bums’ and ‘poo’ as ‘socially embarrassing’, and would not want to ‘advertise it [colonoscopy]’.

### 4.2 Comparison with existing literature

This study is the first to explore barriers and facilitators to colonoscopy among British ethnic minority groups, in their first language. The results of this study are broadly consistent with those exploring the barriers experienced by British ethnic minority groups in other areas of healthcare. For example, a recent systematic review of studies exploring barriers to breast cancer screening, experienced by British ethnic minority groups, reported similar themes, including: ‘Knowledge‐related factors’, ‘Access‐related factors’ and ‘Cultural‐related factors’.^21^

The results of this study contrast, however, with studies exploring barriers to colonoscopy, among ethnic minority groups, in other countries. For example, a review of studies exploring barriers to colonoscopy, among African‐American adults, found that African‐American men frequently reported the invasiveness of the procedure as ‘an affront to their masculinity’,^22^ which was not a barrier that was discussed by any of the participants included in this study (including those of Black ethnicity).

The results of this study also appear to contrast with national trends in colonoscopy attendance, which indicate that ethnic minority groups are unlikely to attend colonoscopy,^6^ despite receiving a medical recommendation from the NHS (see Locus of control). One possible explanation for this discrepancy is that other barriers to colonoscopy, such as language barriers, prohibit attendance (several participants discussed needing a family member to translate the invitation for them, and that the appointment date may pass by the time they are able to arrange this). Another possible explanation is that the medical recommendation comes from the screening programme, and not the individual's general practitioner, and may have less influence/credibility as a result.

Finally, the results of this study are consistent with previous research exploring differences in locus of control between ethnic groups. For example, research comparing locus of control scores between White British, South Asian and Black Caribbean women, found South Asian women scored higher on measures of external locus of control, and concluded that high religiousness may explain some of this variation.^23^

### 4.3 Policy implications and future research

This study has several implications for future research. First, there is a need to validate the findings in an ecological sample (i.e. patients who have had a positive screening result and declined colonoscopy). This has been attempted previously in the UK, without success (colonoscopy decliners were unwilling to meet with researchers to discuss their decision not to attend colonoscopy).^11^ Innovative approaches to collecting these data, therefore, may be required. One possible approach would be to record the pre‐colonoscopy consultation in which patients decide whether to attend colonoscopy (although this would not capture the barriers for those who do not attend the pre‐colonoscopy assessment, which is a minority of colonoscopy non‐attenders).^6^ Such approaches have been employed in other areas of healthcare, and provided valuable insights into patient‐doctor decision‐making.^24^

In addition to the above, quantitative research is needed to understand how barriers and facilitators interact with one another, and which of the perceived barriers and facilitators are predictive of non‐attendance at colonoscopy. This could be achieved through national surveys, distributed to both attenders and non‐attenders, which may be more acceptable to non‐attenders than interviews (a similar approach has been used previously for flexible sigmoidoscopy screening non‐attenders, and successfully quantified the barriers for this).^25^

This study also has several implications for policy. First, to reduce barriers to screening (and colonoscopy), there is strong public health mandate to update national systems, so that invitations are sent in the first language of recipients (these data are recorded on the GP clinical systems, from which national screening programmes obtain the necessary information to administer invitations [although their current access to these systems does not allow them to access information about a patient's first language and, therefore, need to be amended]). Second, there is a need to address cultural taboos and stigma, surrounding cancer, the colonoscopy procedure, and other sensitive topics, in order to make access to screening and colonoscopy further equitable still (this could be achieved through changes to the invitation materials, which, as discussed, could be tailored to patients' first language). Finally, there is a wider need to educate the population that bowel cancer can be a treatable disease, particularly when it is diagnosed early, and that participating in screening (and attending follow‐up colonoscopy) can improve patient outcomes (this could also be achieved through changes to the invitation letter, as well as social media campaigns, which could be targeted according to the individual's first language).

### 4.4 Study limitations

This study has several limitations. First, it is subject to hypothetical bias, as several participants had not received an abnormal bowel cancer screening result, but rather, were asked to imagine they had. Second, all of the Black African participants were Somali. As such, this study did not investigate the views of adults of other Black African nationality, who may experience different cultural barriers to attending colonoscopy (for example, 7% of Muslims in Kenya are Shia Muslims, while only 2% of Muslims in Somalia are Shia Muslims [the majority being Sunni Muslims]).^26^ Third, member checking was not conducted, as the researchers were not aware of the identities of participants recruited by Agroni (member checking is not always necessary; however, given that the authors were interpreting transcripts of interviews, which had been translated from multiple languages, member checking might have been particularly valuable in the present study, to validate the responses of the participants, and the authors' interpretations thereof). Fourth, all but one participant was from England, and there may be contextual differences between Scotland, Wales and Northern Ireland, not identified in this research. Fifth, all interviews were conducted online and over the phone, which may have prevented some populations from being able to participate, and their views excluded from the data (i.e. those without internet or a telephone contract). Sixth, different recruitment strategies were employed to recruit White British participants and participants from South Asian and Black ethnic groups, and they may not be directly comparable as a result. Finally, participants were not asked about their migration, socioeconomic or education status, which may have added important contextual data about the participants' responses, and possible differences between participants recruited via Agroni and social media (previous research suggests that locus of control is strongly related to health literacy,^27^ educational attainment,^27^ and a range of other socio‐demographic factors^27^, ^28^). Measuring these factors may have aided our interpretation of the data, and explained how religious beliefs manifest as barriers in some participants, and facilitators in others.

## 5 CONCLUSION

The results imply that South Asian, Black African and Black Caribbean adults experience unique barriers to attending colonoscopy (offered in the context of FIT‐based screening for CRC), which are different to those experienced by White British adults. Further, the results suggest that the decision to attend colonoscopy is often influenced by the family members and religious beliefs of these groups. Person‐centred approaches, designed with these points in mind, may help to address cultural barriers to colonoscopy and ensure equitable uptake.

## AUTHOR CONTRIBUTIONS

**Robert S. Kerrison**: Conceptualization; methodology; data collection; formal analysis; writing—original draft; writing—review & editing; supervision; funding acquisition. **Natalie Gil**: Data collection; Formal analysis; writing—original draft; writing—review & editing. **Elizabeth Travis**: Formal analysis; writing—original draft; writing—review & editing. **Robyn Jones**: Conceptualization; methodology; writing—original draft; writing—review & editing. **Katriina L. Whitaker**: Conceptualization; methodology; writing—review & editing; funding acquisition. **Colin Rees**: Conceptualization; methodology; writing—review & editing; funding acquisition. **Stephen Duffy**: Conceptualization; methodology; writing—review & editing; funding acquisition. **Christian von Wagner**: Conceptualization; methodology; writing—review & editing; supervision; funding acquisition.

## CONFLICT OF INTEREST STATEMENT

None to declare.

## ETHICS STATEMENT

The study was approved by University College London's Research Ethics Committee: 5299/003.

## Supporting information

Supporting Information S1

Supporting Information S2

Supporting Information S3

Supporting Information S4

Supporting Information S5

## Data Availability

All data files are available via Open Science Framework (https://osf.io/pvk3w/).
