# Supplementary material for: Barriers to colonoscopy in UK colorectal cancer screening programmes: Qualitative interviews with ethnic minority groups
Source: Psychooncology. 2023 Apr 6;32(5):779–92. doi: 10.1002/pon.6123 (PMC10946452; doi:10.1002/pon.6123)
Supplement: Supplementary file 1 — Supporting Information S1 [file PON-32-779-s002.docx]

**Appendix A. Pre-Interview Questionnaire.**

1. What is your gender?
2. What is your ethnicity?
3. Where do you live (name of city / town)?
4. What is your main language?
5. What is your religion (if any)?
6. Have you ever been invited to take part in the Bowel Cancer Screening Programme?
7. When you were invited to take part in the Bowel Cancer Screening, did you complete the test kit?
