# Supplementary material for: Barriers to colonoscopy in UK colorectal cancer screening programmes: Qualitative interviews with ethnic minority groups
Source: Psychooncology. 2023 Apr 6;32(5):779–92. doi: 10.1002/pon.6123 (PMC10946452; doi:10.1002/pon.6123)
Supplement: Supplementary file 2 — Supporting Information S2 [file PON-32-779-s004.docx]

**Appendix B. Public attitudes towards colonoscopy as a follow-up test for positive bowel cancer screening results: an online and telephone interview study**

**(PI: Dr. Robert Kerrison)**

OBJECTIVES

- To explore public attitudes towards colonoscopy as a follow-up test for positive bowel cancer screening results.

METHODS

- Online and telephone interviews (60 minutes each) with members of the public who have previously taken part in bowel cancer screening.
- Interviews to take place online via videocall (e.g. Skype) or over the telephone (depending on the participant’s preference).

PARTICIPANTS

- Men and women, aged 60-74 years, who have previously taken part in bowel cancer screening.
- Recruited by Agroni / local community centres located in socioeconomically deprived areas with low colonoscopy uptake.

**INTRODUCTIONS**

- Dr. Robert Kerrison, Senior Research Fellow at University College London

**IDENTITY CONFIRMATION**

- Confirm that the name of the interviewee matches the name on record.

**PURPOSE OF THE STUDY**

- Explore public attitudes towards colonoscopy as a follow-up test for positive bowel cancer screening results.
- Follow-up rates for bowel cancer screening are currently 85%, which is lower than ideal.
- We know that there are many factors contributing to these rates, including patient ethnicity and socioeconomic deprivation. We would like to better understand these factors to enable us to find ways to improve follow-up where appropriate.
- The results will inform the development of public health strategies to improve attendance at colonoscopy in the future.

**EXPLANATION OF THE INTERVIEW**

- I’d like to ask you some questions about you generally, your participation in cancer screening in the past, and your thoughts on why some patients do not attend colonoscopy after receiving a positive bowel cancer screening results.
- The interview should take about **60 minutes.**
- **I’ll be audio recording** our conversation and will transcribe it afterward, so that I can be sure I have an accurate record of the interview.
- **No identifying** information about you will be included in the transcripts, or in any reports, publications or presentations that arise from the interview.

**INFORMED CONSENT**

- Provide verbal summary of consent form.
- **Ask participant if he/she has any questions; address questions, if any.**

**QUALITATIVE DISCUSSION**

1. **About you**
2. So, to begin with, can you tell me a bit about yourself?

- Are you currently employed? What did you do before you retired?
- Have you always lived in <Area>?
- What are some of your hobbies? Do you attend any community groups?

1. **About the area**
2. Thank you, that is very interesting. Could you also tell me a bit about <Area>?

- What are some of the things you like about it?
- Is there anything you don’t like about it?

1. And in terms of the local hospital, could you tell me about that?

- Where is the hospital located?
- Is it easy for you to get to?
- How would you get there? How long would it take?
  1. If they say ‘**drive**’:
     1. What’s the car parking like?
     2. Is it easy to find a space? Is it expensive?
  2. If they say ‘**relative/friend**’
     1. Who?
     2. Would this make it difficult to attend the appointment at the date and time offered?
  3. If they say ‘**hospital transport**’
     1. Is this easy to arrange? Have you previously used this?
     2. Is it reliable?
  4. If they say ‘**Public transport**’
     1. What’s the public transport like?
     2. Is it reliable? Is it very expensive?

1. Apart from the Hospital, are there any other healthcare services that you use?
   1. If yes. What other services do you use?
      1. Do you see them often / What is your relationship with them like?
      2. In general, do you follow their medical advice? What aspects make you more/less at ease?
2. **Previous experience with bowel cancer screening**
3. Great, thank you for that. I’d like to move on now to discuss the bowel cancer screening programme. I understand you’ve been invited and taken part in the past, is that correct?

- Do you know how the test works? What it’s looking for?
- If your next test came back as ‘positive’, what would you take that to mean?
- Do you know what the next steps would be? What tests you might need to do?

1. (That’s right) If your next test was positive, you would be invited to meet with a nurse to discuss the need for a test called a ‘colonoscopy’.

- In your own words, can you tell me what you understand a colonoscopy to be?
- Have you previously had a colonoscopy? Do you know anyone who has?
  1. If yes to either: what was your / their experience of the test?

**IV. First activity**

1. Thank you. For this next bit, I want you to imagine that you have recently taken part in the bowel cancer screening programme and have just received this letter in the post. Please take a couple of minutes to read the letter. You can do this out loud or in your head. Once you’ve finished reading the letter, I’ll ask you some questions about your initial thoughts.

- (Agroni interviews only) If the participant cannot read English, provide them with a translated copy and ask what they would do if they received the English language letter, would they have a family member interpret, call the helpline, etc.
- How do you think you would feel if you received this letter?
- What would you do after reading this letter?
  1. Would you discuss it with anyone?
  2. **If yes:** Who?
  3. **If no:** Is there a particular reason for this?
- Would you try and find out more about bowel cancer?
  1. **If yes:** Where would you first look? GP, NHS website, internet, charities, news outlets?
- Would you be able to attend the appointment day and time offered in the letter?
  1. **If no:** Why would you be unable to attend this appointment?
  2. Do you have other commitments during the week that would make it difficult for you to schedule a new appointment?
  3. **If yes:** What if the appointment were on another day of the week? All appointments take place between 9am and 5pm, Monday to Friday.
- Realistically, do you think you would go to the appointment?
  1. **If no:** Can you tell me why? Do you have any concerns?
  2. **If yes:** Are there any circumstances under which you think you would not be able to attend or would need to cancel?
- Let’s imagine you were able to attend your appointment. Would you go by yourself or would you take someone with you?
  1. Who would you take with you?
  2. Why would you take <person> with you? What role would they serve?
- (Agroni interviews only) Would you make use of the interpreter service mentioned in the letter?
  1. Are there any other facilities you can think of that would be useful?
- As the letter explains, the purpose of the appointment is to determine whether you are fit and healthy enough to undergo colonoscopy, and to provide you with an opportunity to ask questions and determine whether colonoscopy is something you’d like to do.
  1. Is there any reason why you think you might not be eligible for colonoscopy?
     1. Do you have any existing health conditions?
  2. What are some of the things about the procedure you would want the nurse to tell you about before you make a decision?
     1. Is there anything you’d be particularly concerned about?

**Second activity**

1. That’s great, thank you. I’ll now provide you with a short description of the colonoscopy procedure, to give you a bit more of a sense of what it involves.

- After hearing that description of the test, do you have any concerns about the test?
  - What about the bowel preparation? Would you be able to do that do you think?
  - And what about in relation to COVID-19. Would you have any concerns about doing the test because of that?
    - **If yes:** Can you explain this in more detail?
    - **If no:** What if you were told that you and anyone in your household would have to self-isolate three days prior to the day of your colonoscopy? Would that present any issues for you or your household?
- And after hearing that description of the test, do you think your family and friends would want you / not want you to do the test?
  - Is there anyone who would really want you / not want you to go for colonoscopy?
    - If yes, Who? Can you explain why in more detail?
- Colonoscopy, bowels and bowel cancer can be sensitive topics. Do you think this is true for you or your family and friends?
  - If yes: Can you explain this in more detail? Are these concerns shared by others?
- Culturally, is there anything else you think might present an issue?
  - For example, Jehovah Witnesses are unable to accept blood product for religious reasons. Is there anything like that which might present an issue?
- Based on what you’ve heard today, do you think you would go for colonoscopy if your next screening test result came back positive?
  - **If yes:** what would be your main reasons for having the test?
    - Even though you said you would have the test, do you have any concerns about it at all?
  - **If no:** What would be your main reasons for not having the test?
    - Even though you said you wouldn’t have the test, is there anything that makes you question your decision.?

1. **Interventions for system improvement**
2. Great. Just a few more questions:

- What do you think could be done to improve the total number of people who attend colonoscopy following an abnormal bowel cancer screening result?
- (Agroni interviews only) What do you think could be done to improve the total number of <Insert population characteristic> patients who attend colonoscopy, specifically.
