# Supplementary figures and images for: Barriers to colonoscopy in UK colorectal cancer screening programmes: Qualitative interviews with ethnic minority groups
Source: Psychooncology. 2023 Apr 6;32(5):779–92. doi: 10.1002/pon.6123 (PMC10946452; doi:10.1002/pon.6123)

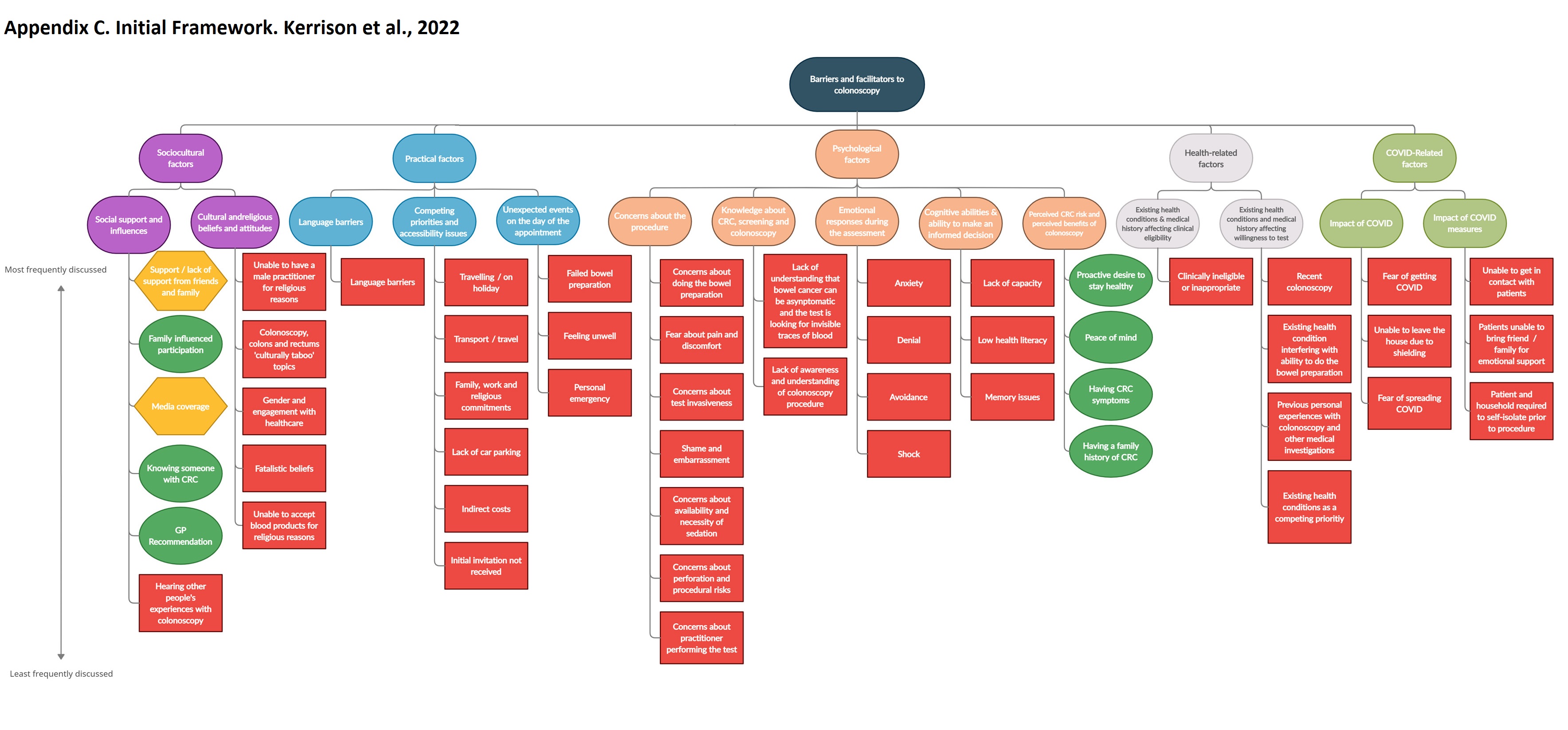

Supplement: Supplementary file 3 — Supporting Information S3 [file PON-32-779-s001.jpg]
